# Bone bruise in anterior cruciate ligament rupture entails a more severe joint damage affecting joint degenerative progression

**DOI:** 10.1007/s00167-018-4993-4

**Published:** 2018-06-05

**Authors:** Giuseppe Filardo, Luca Andriolo, Giorgio di Laura Frattura, Francesca Napoli, Stefano Zaffagnini, Christian Candrian

**Affiliations:** 1 II Orthopaedic and Traumatologic Clinic, Rizzoli Orthopaedic Institute, Via di Barbiano, 1/10, Via G. C. Pupilli, 1, 40136 Bologna, Italy; 2 Ospedale Regionale di Lugano, EOC, Via Tesserete, 46, Lugano, Switzerland; 3 Nano-Biotechnology Laboratory, Rizzoli Orthopaedic Institute, Via di Barbiano, 1/10, Bologna, Italy

**Keywords:** Bone bruise, Bone contusion, ACL, Knee

## Abstract

**Purpose:**

During anterior cruciate ligament (ACL) injury, the large external forces responsible for ligament rupture cause a violent impact between tibial and femoral articular cartilage, which is transferred to bone resulting in bone bruise detectable at MRI. Several aspects remain controversial and await evidence on how this MRI finding should be managed while addressing the ligament lesion. Thus, the aim of the present review was to document the evidence of all available literature on the role of bone bruise associated with ACL lesions.

**Methods:**

A systematic review of the literature was performed on bone bruise associated with ACL injury. The search was conducted in September 2017 on three medical electronic databases: PubMed, Web of Science, and the Cochrane Collaboration. Preferred Reporting Items for Systematic Reviews and Meta-analysis (PRISMA) guidelines were used. Relevant articles were studied to investigate three main aspects: prevalence and progression of bone bruise associated with ACL lesions, its impact on the knee in terms of lesion severity and joint degeneration progression over time and, finally, the influence of bone bruise on patient prognosis in terms of clinical outcome.

**Results:**

The search identified 415 records and, after an initial screening according to the inclusion/exclusion criteria, 83 papers were used for analysis, involving a total of 10,047 patients. Bone bruise has a high prevalence (78% in the most recent papers), with distinct patterns related to the mechanism of injury. This MRI finding is detectable only in a minority of cases the first few months after trauma, but its presence and persistence have been correlated to a more severe joint damage that may affect the degenerative progression of the entire joint, with recent evidence suggesting possible effects on long-term clinical outcome.

**Conclusion:**

This systematic review of the literature documented a growing interest on bone bruise associated with ACL injury, highlighting aspects which could provide to orthopaedic surgeons evidence-based suggestions in terms of clinical relevance when dealing with patients affected by bone bruise following ACL injury. However, prospective long-term studies are needed to better understand the natural history of bone bruise, identifying prognostic factors and targets of specific treatments that should be developed in light of the overall joint derangements accompanying ACL lesions.

**Levels of evidence:**

IV, Systematic review of level I–IV studies.

## Introduction

The large external forces responsible for anterior cruciate ligament (ACL) rupture also cause a violent impact between tibial and femoral articular cartilage, which is transferred to bone and results in bone bruise [60, 70, 80]. Such MRI finding is best diagnosed on fluid-sensitive sequences such as T2-weighted images showing increased signal intensity, with or without decreased signal intensity on T1-weighted images. In addition, short tau inversion recovery (STIR) sequences can provide more sensitive information by suppressing the signal from normal medullary fat [55, 62]. Sensitivity and specificity of MRI detection have already been documented to be 83/96 and 86/96%, respectively. Moreover, histological studies allowed to correlate these MRI findings to tissue alterations, including microfracture of the subarticular spongiosa, with osteocyte necrosis and empty lacunae, bleeding in the fatty marrow and edema [55, 62]. Bone bruise associated with ACL rupture has been extensively investigated [62, 63], but several aspects remain controversial and await evidence on how this MRI finding should be managed while addressing the ligament lesion.

The aim of this systematic review was to document the available evidence on bone bruise associated with ACL lesions, investigating its prevalence and progression, as well as the impact on joint and prognosis, with the hypothesis that bone bruise can influence knee degeneration and patient clinical outcome. This would provide orthopaedic surgeons with evidence-based suggestions in terms of clinical relevance when dealing with patients affected by bone bruise following ACL injury.

## Materials and methods

**Fig. 1:**
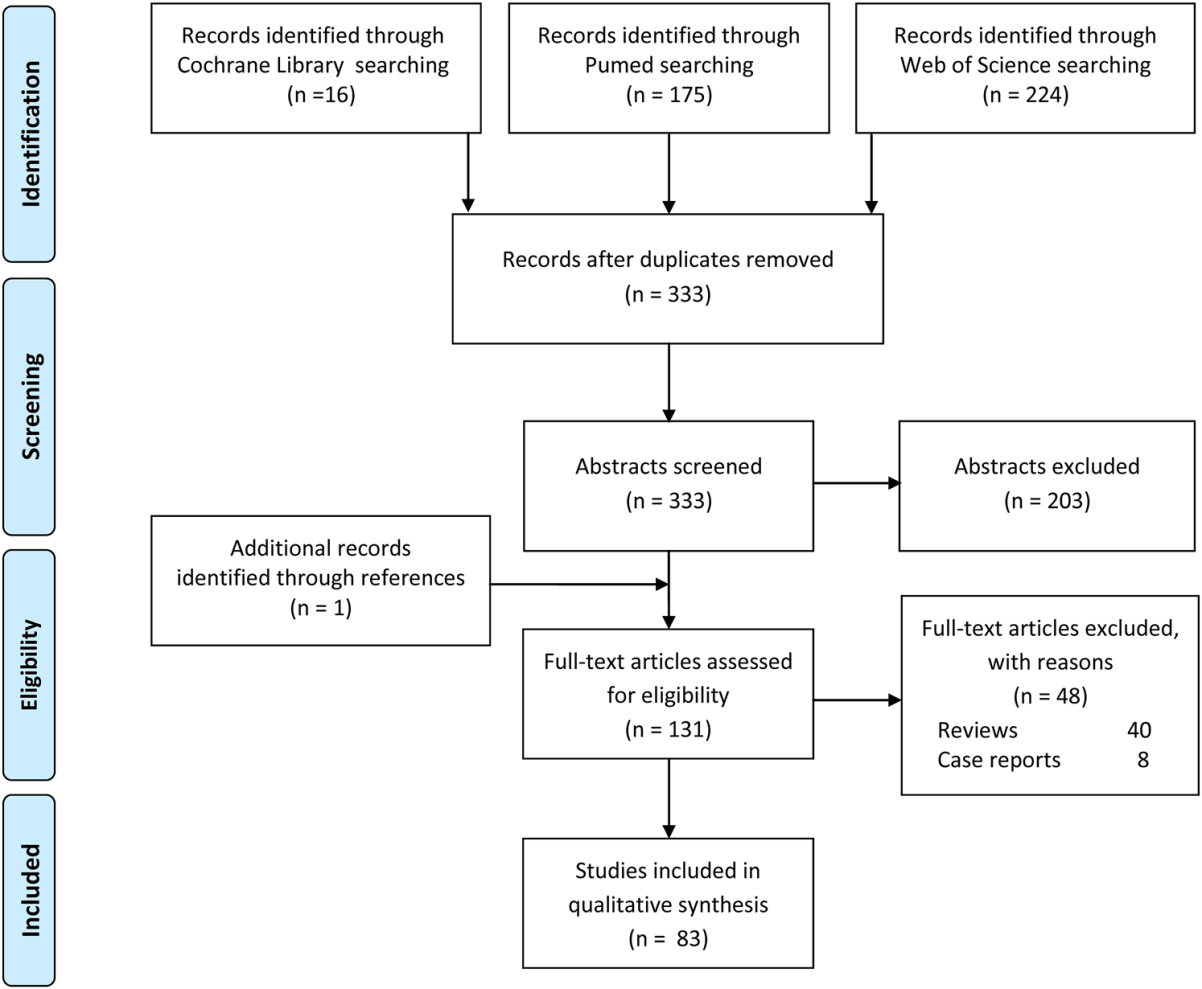
PRISMA flowchart of the systematic literature review

A systematic review of the literature was performed on bone bruise associated with ACL injury. This search was conducted on September 4th, 2017, using the following string on three medical electronic databases, PubMed, Web of Science, and the Cochrane Collaboration: [(subchondral edema) OR (bone bruise) OR (bone marrow edema) OR (bone marrow lesion) OR (bone contusion)] AND [(ACL) OR (anterior cruciate ligament)]. The Preferred Reporting Items for Systematic Reviews and Meta-analysis (PRISMA) guidelines were used [65] (Fig. 1). Two independent authors separately performed the screening process according to preset inclusion and exclusion criteria, study analysis and data tabulation. A final literature summary was obtained by consensus, with disagreements solved by discussion with a third reviewer (GdLF, FN and GF).

First, articles were screened by title and abstract according to the following inclusion criteria: clinical reports of any level of evidence, written in English language, with no time limitation, on the association of bone bruise with ACL lesions. Exclusion criteria were articles written in other languages, preclinical or ex vivo studies, reviews, case reports or clinical studies not evaluating prevalence, progression and impact on the joint and on prognosis. Second, the full texts of the selected articles were screened, with further exclusions according to the previously described criteria. Reference lists from the selected papers were also screened. Relevant data (type of study, no of patients and demographics, injury–MRI time and sequence, follow-up, edema size/grading, edema distribution, prevalence and progression, correlation with other joint lesions and prognosis) were then extracted and collected in a unique database to be analysed for the purposes of the present manuscript. All relevant articles included in this systematic review were studied to investigate three main aspects: the prevalence and progression of bone bruise associated with ACL lesions, its impact on the knee in terms of lesion severity and progression of joint degeneration over time and, finally, the influence of bone bruise on patient prognosis in terms of clinical outcome.

## Results

**Fig. 2:**
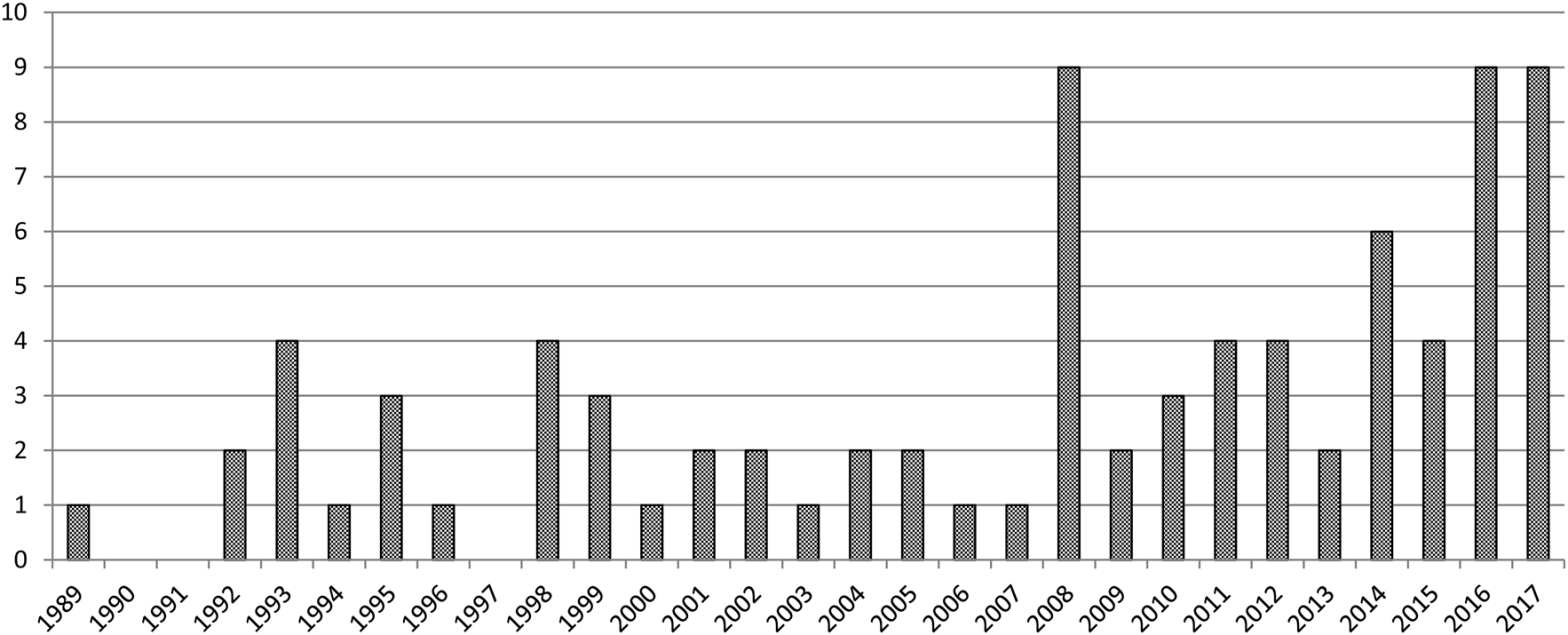
The analysis of publications per year shows growing interest on bone bruise in ACL lesions with an increasing number of published studies over time

This systematic review underlined a growing interest on this topic, with an increasing number of papers published over time, more than half in the last 10 years (Fig. 2). The database search identified 83 papers used for the analysis (a detailed study description is reported in Table 1; Fig. 1).

**Table 1:** Detailed description of the 83 studies selected in this systematic review

References	Type of study	No. of pts, sex, age, mean (range)	MRI–injury time sequences	Follow-up (months)	Edema size grading	Edema distribution	Prevalence and progression		Correlation with other joint lesions and prognosis
Mink [64]	NR	25, NR, NR	≤ 2 weeks, 1.5 T, seq T1	2	NR	NR	72%	NR	NR
Cobby [14]	NR	103, 75 M, 28 F, 38 (15–70)	NR, 1.5T, seq NS	NR	NR	NR	NR	NR	NR
Speer [76]	Retrospective	54, 30 M, 24 F, 28 (14–44)	≤ 45 dd, 1.5 T, seq T1, spin density, and T2	NR	NR	NR	83%	NR	NR
Graf [39]	NR	98, NR, NR	≤ 6 weeks; 7 weeks–6 mm; >6 mm, 1.5 T, seq T1 and T2	NR	Area	30 LTP, 38 LFC 12 MTP, 9 MFC	48%	NR	NR
Spindler [78]	Prospective	54, NR, 24.5	≤ 3 mm, 1.0 or 1.5 T, seq T1, T2 and GE	NR	NR	29 LTP, 37 LFC 9 MTP, 3 MFC	80%	NR	Osteochondral
Tung [88]	Retrospective	99, 62 M, 37 F, 31 (14–47)	≤ 20.5 and 16.9 weeks, 1.5 and 1.0 T, seq T1, T2 and PD	NR	NR	15 LTP, 13 LFC 7 MFC	26%	NR	NR
Nawata [66]	Retrospective	56, 26 M, 30 F, 28 (13–59)	≤ 1; 1–12;≥ 12 mm, 1.0 and 1.5 T, seq T2, and PD	NR	NR	17 LTP, 19 LFC 1 MFC	36%	NR	NR
Gentili [37]	Retrospective	89, 62 M, 27 F, 30 (16–75)	≤ 1; 1–3; > 3 mm. 1.5 T, seq T2	NR	NR	NR	NR	NR	NR
Speer [77]	Retrospective	42, 20 M, 22 F, 32 (16–58)	≤ 1 mm, 0.35, 0.50 and 1.5 T, seq T1, T2 SE and GE	NR	Area	34 LTP, 17 LFC 12 MTP, 4 MFC	NR	NR	NR
Stein [79]	Retrospective	20, 10 M, 10 F, 24 (13–48)	≤ 4 dd(1 to 23 dd), 1.5 T, seq PD and T2	40 (24 to 73)	NR	20 LTP, 13 LFC 3 MTP, 1 PAT	100%	NR	NR
Zeiss [102]	Retrospective	71, 37 M, 34 F, (14–36)	≤ 1 mm, 1.5 T, seq GE, T1 and T2	NR	NR	NR	36.60%	NR	NR
Brandser [9]	Retrospective	74, NR, NR	≤ 6 weeks, 1.5 T, seq PD, T2 and T1	NR	NR	NR	NR	NR	NR
Yeung [98]	NR	16, 10 M, 6 F, 25 (13–43)	6 dd to 3 y, 0.5 and 1.5 T, seq T1, SE, GE, T2, PD, T1 TSE and TFE	NR	NR	NR	NR	NR	NR
Johnson [48]	Prospective	10, 10 M, 21 (15–36)	≤ 2 weeks, 1.5 T, seq T1	NR	NR	NR	NR	NR	NR
Dimond [23]	Retrospective	87, 50 M, 37 F, 29 (16–43)	≤ 6 weeks, seq T1, T2 and PD	NR	NR	30 LTP, 24 LFC 4 MTP, 11 MFC	68%	NR	NR
Lahm [56]	Prospective	38, NR, 31	NR, seq T1, T2 and STIR	5.5 and 40.8	NR	NR	62%	NR	NR
Faber [27]	Retrospective	23, 18 M, 5 F, 30 (20–49)	≤ 12 dd 1.5 T, seq T1 and T2 FSE	72	NR	23 LTP, 23 LFC	NR	65%	NR
Kaplan[49]	Retrospective	25, 20 M, 5 F, 28 (16–52)	≤ 4 weeks, 1.5 T, seq T1, GRE, WE DESS and STIR	NR	NR	25 LTP, 24 LFC	NR	NR	NR
Lee [58]	Retrospective	19, 5 M, 14 F, (5–16)	≤ 2; 2–8;> 8 weeks, 1.5 T, seq T1 and T2	NR	NR	NR	68%	NR	NR
Johnson [47]	Prospective	40, NR, 18 (15–23)	≤ 1 week, NR, seq NS	0.2, 0.5, 0.7 and 1	NR	NR	NR	NR	NR
Costa-Paz [16]	Cohort study	21, 15 M, 6 F, 31 (20–58)	NR	34	NR	11 LTP, 16 LFC 1 MTP, 1 MFC	NR	NR	NR
Fang [28]	Prospective	12, 9 M, 3 F, 18 (17–23)	NR	NR	NR	12 LTP, 12 LFC	NR	NR	NR
Bretlau [10]	Prospective	64, 33 M, 31 F, 36 (15–68)	≤ 5 dd, 0.1 T, seq T2, PD 3D-GE and STIR	4 and 12	NR	13 LTP, 8 LFC 8 MTP, 5 MFC	63%	NR	NR
Chen [12]	Retrospective	32, 22 M, 10 F, 29	< 6; > 6 weeks, 1.5 T, seq T1, T, GE and PD	NR	NR	NR	63%	NR	NR
Fayad [29]	Retrospective	84, 42 M, 42 F, (16–39)	NS, 1.5 T, seq T1, T2, T2 FS and FSE	NR	NR	62 LTP, 50 LFC 35 MTP, 4 MFC	NR	NR	NR
Davies [19]	Prospective	30, 16 M, 14 F, 28 (17–39)	≤ 3.4 weeks, 1.0 T, seq T1, GE and STIR	3,2	Volume	NR	NR	NR	Osteochondral
Terzidis [83]	NR	255, 197 M, 58 F, 24	≤ 1; 2–4 mm, 1.0 T, seq T1 SE, T2 and STIR	31	NR	4 LTP, 29 LFC 4 MTP,5 MFC	NR	NR	NR
Tiderius [87]	NR	24, 14 M, 10 F, 27 (17–40)	≤ 3 weeks, 1.5 T, seq T1 Gd	NR	Area	6 LTP, 15 LFC	96%	NR	Osteochondral
Fithian [33]	Prospective	209, 101 M, 108 F, 39 (16–69)	≤ 4 weeks, 1.5 T, seq NR	79	NR	NR	53%	NR	NR
Vincken [91]	Prospective	664, 460 M, 204 F, (16–45)	≤ 4 weeks, 0.5 T, seq DSE and 3D T1 GE	6	NR	43 LTP, 44 LFC 31 MTP, 7 MFC 6 PAT	18.70%	NR	LM MCL–LCL
Wu [97]	Pilot study	52, 22 M, 30 F, NR	NS 1.0 T, seq FSE, FS T1, GE, T1 FSE and T2 FS FSE	NR	Beattie and colleagues	NR	7.70%	NR	NR
Hernandez-Molina [44]	NR	258, 148 M, 110 F, 67	NR, 1.5 T, seq SE PD, T2, SE, FS and PD	15 and 30	NR	NR	53.80%	NR	Osteochondral
Nishimori [67]	NR	39, 25 M, 14 F, 23 (14–55)	≤ 8 dd, 0.3 T, seq SE PD and T2	NR	NR	NR	89.70%	NR	Osteochondral LM
Collins [15]	Retrospective	48, 26 M, 22 F, 29	≤ 6 mm, 1.5 T, seq FSE PD, FS FSE T2, T2 GRE, CSE PD and CSE T2	NR	NR	NR	33%	NR	NR
Hanypsiak [41]	Cohort study	54, NR, NR	≤ 3 weeks, 1.0 T, seq T1, PD, T2, T2 FS and FLASH	153 (141–165)	NR	29 LTP, 37 LFC 9 MTP, 3 MFC	80%	0%	NR
Atkinson [5]	Retrospective	1546, NR, NR	0–4, 4–10, 10–26 and 26–52 weeks, 1.5 T, seq multiplanar FS	NR	NR	NR	NR	NR	MM LM MCL
Viskontas [92]	Prospective	100, 69 M, 31 F, 29 (13–61)	≤ 6 weeks, 1.5 T, seq FS and FSE	NR	Area ICRS	NR	NR	NR	NR
Frobell [36]	RCT	121, 89 M, 32 F, 26	≤ 19 ± 6.5 dd, 1.5 T, seq 3D-FLASH, T2 3D-GRE, DETSE and STIR	NR	Volume	NR	98%	NR	Cortical depression fractures
Bolbos [8]	Retrospective	31, 22 M, 9 F, 31	≤ 2 mm, 3 T, seq 2D T2 FS FSE and 3D-SPGR	NR	Area	13 LTP, 9 LFC	93%	NR	Osteochondral
Frobell [35]	NR	58, 42 M, 16 F, 26	≤ 5 weeks, 1.5 T, seq 3D-FLASH, T2 3D-GRE, DETSE and STIR	3, 6 and 12	Volume	NR	100%	NR	NR
Xiaojuan [59]	Prospective	38, 28 M, 10 F, 35 (20–66)	≤ 2 mm, 3 T, seq T2 FS FSE, SPGR and 3D MRSI	NR	Volume	NR	NR	NR	Osteochondral
Halinen [40]	Prospective	44, 19 M, 25 F, 39 (21–64)	NS, 0.23 T or 1.5 T, seq T1, T2, FSE, DE, SE, FS, PD FSE, PD SE and STIR	NR	NR	NR	88.60%	NR	NR
Yoon [99]	Retrospective	145, 124 M, 21 F, 31 (10–56)	≤ 6 weeks; 1.5–3 mm; 3–12 mm; >12 mm, 1.5 T, seq T1, T2 FS, PD, PD DE and FS	NR	Area	NR	60.70%	NR	NR
Dunn [26]	Prospective	525, 304 M, 221 F, 23	NR	NR	NR	NR	NR	NR	NR
Quelard [73]	Prospective	217, 139 M, 78 F, 29 (14–62)	≤ 6 mm, NR	1.5 and 3	NR	156 LTP, 104 LFC	72%	NR	NR
Theologis [84]	Cohort study	9, 5 M, 4 F, 35 (27–45)	≤ 8 weeks, 3 T, seq T2 FS FSE	0.5, 6 and 12	Volume	NR	NR	NR	Osteochondral
Frobell [34]	NR	61, 45 M, 16 F, 26	NR, 1.5 T, seq FLASH, T2, DETSE and STIR	3, 6, 12 and 24	Volume	58 LTP, 47 LFC	NR	NR	NR
Yoon [101]	Retrospective	80, 58 M, 22 F, 30	≤ 8 dd, 1.5 T, seq T1 and T2	NR	NR	50 LTP, 46 LFC 18 MTP, 16 MFC	84%	NR	MM LM
Jelić [46]	NR	120, 88 M, 32 F, 31	≤ 1 mm, 0.3 T, seq SE T1W1, FS T2W1 and STIR	NR	NR	18 LFC, 4 MFC	33%	NR	MM LM
Potter [72]	Prospective	40, 16 M, 24 F, 37 (15–53)	≤ 8 weeks, 1.5 T, seq T2 and CPMG	132	Area	36 LTP, 30 LFC	NR	NR	Osteochondral
Van Dyck [89]	Retrospective	97, 62 M, 35 F, 49 (12–81)	≤ 6 weeks, 1.5 and 3.0 T, seq FS TSE WI, SE T1 WI, TSE PD and T2 WI	NR	NR	NR	41%	NR	NR
Kijowski [50]	Retrospective	114, 57 M, 57 F, 26	≤ 3 weeks, 1.5 and 3.0 T, seq T2 FS FSE and FSE	12	Volume	106 LTP, 88 LFC 57 MTP, 30 MFC	96%	NR	Cortical fractures
Szkopek [81]	Prospective	17, 10 M, 7 F, 28 (23–34)	≤ 2 dd, 1.5 T, seq T2 FS, T1 and 3D Ge T2	0.5, 1 and 2	Volume	NR	NR	NR	NR
Yoon [100]	Retrospective	151, 130 M, 21 F, 31 (10–56)	≤ 6 weeks; 6 weeks − 3 mm; 3–12 mm;> 12 mm, 1.5 T, seq T1, T2 FS, PD, PD DE and FS	NR	Area	NR	NR	NR	NR
Bisson [7]	Case control	171, 89 M, 82 F, NR	≤ 6 weeks, NR, seq NR	NR	Volume Area	145 LTP, 132 LFC 44 MTP, 11 MFC	90%	NR	LM
Roemer [74]	NR	62, 50 M, 12 F, 26	NS, 1.5 T, seq 3D FLASH, T2 GRE, DETSE and STIR	12, 24, 36, 48, 60 and 72	Volume	NR	NR	NR	NR
Chang [11]	Retrospective	154, 130 M, 24 F, 32 (14–56)	< 3, > 3 mm, 1.5 T, seq FS T2, T1 and PD	NR	NR	NR	NR	NR	NR
Wittstein [96]	Case series	73, 28 M, 45 F, 16	≤ 6 weeks, 1.5 T, seq FS FSE T2	NR	Volume	67 LTP, 70 LFC 45 MTP, 31 MFC	NR	NR	NR
Wissman [94]	Retrospective	7, 5 M, 2 F, NS	NR, 1.5–3 T, seq T2, FS FSE and PD	NR	NR	4 LTP, 4 LFC 4 MTP, 4 MFC	NR	NR	NR
Illingworth [45]	Retrospective	50, 26 M, 24 F, 19	≤ 30 dd, 1.5 T, seq T2 TSE FS, PD TSE, T1 SE and TSE PD	NR	Volume Area	NR	86%	NR	Menisci
Chin [13]	Retrospective	88, 72 M, 16 F, 27	≤ 10 weeks, 1.5 T, seq T1 and T2	NR	NR	37 LTP, 43 LFC 23 MTP, 16 MFC, 4 PAT	65.90%	NR	NR
Wissman [95]	Retrospective	132, 82 M, 50 F, 30 (14–70)	≤ 4 weeks, 1.5 or 3 T, seq T2, FSE and PD	NR	NR	NR	NR	NR	NR
Culvenor [17]	NR	111, 71 M, 40 F, 26 (18–50)	≤ 12 mm, 3 T, seq 3D PD VISTA, PD TSE and STIR	12	Area MOAKS	15 LTP, 10 LFC 9 MTP, 10 MFC 5 PAT, 22 TRO	NR	NR	Osteochondral
Herbst [43]	Retrospective	500, NR, 29	≤ 1 mm, NR, seq NR	NR	Area	396 LTP, 257 LFC	NR	NR	NR
Kim [51]	Retrospective	8, 5 M, 3 F, 23 (16–30)	≤ 1 mm, 1.5 and 3 T, seq FSE	NR	Volume	NR	NR	NR	NR
Filardo [32]	Retrospective	134, 98 M, 36 F, 32	≤ 6 mm, 1.5 T, seq FSE, PD FS and dual FSE	80	Area WORMS	22 LTP, 12 LFC 4 MTP, 4 MFC 2 PAT	55.20%	NR	NR
Pezeshki [71]	Prospective	175, 149 M, 26 F, < 45 (18–45)	≤ 1 mm, 0.3 T, seq T1 and T2 FS, fluid suppression, GE and T2 SE	12	Area	NR	30,90%	NR	MM MCL
Kluczynski [52]	Cross sectional	59, 59 M, 23	≤ 6 weeks, NR, seq NR	NR	NR	48 LTP, 46 LFC 14 MTP, 3 MFC	NR	NR	NR
Ahn [1]	Retrospective	249, 33 M, 216 F, 38 (18–53)	≤ 15.4 ± 15.6 (1–52) and 4.2 ± 5.7 (1–50) weeks, 1.5 T, seq T1, T2 and PD	NR	Area	NR	NR	NR	Meniscal injuries
Culvenor [18]	Prospective	93, 56 M, 37 F, 29	NS, 3 T, seq PD VISTA, PD TSE and STIR	12 and 36	Area	NR	NR	22% (12 mm)	NR
Palmieri-Smith [68]	Prospective	22, 10 M, 12 F, 20	≤ 2 weeks, 3 T, seq FSE and T2	12	Area	10 LTP, 11 LFC 2 PAT	NR	NR	Osteochondral
Kluczynski [53]	Case control	384, 209 M, 175 F, NS	≤ 6 weeks, NS, seq NR	NR	NR	290 LTP, 251 LFC 105 MTP, 27 MFC	NR	NR	NR
Song [75]	Retrospective	193, 141 M, 52 F, 32.3 (15–55)	≤ 6 weeks, 1.5 T, seq FS and T2	NR	Area ICRS	141 LTP, 117 LFC 41 MTP, 12 MFC	NR	NR	LM ALL
Gong [38]	Prospective	54, 31 M, 23 F, 30	≤ 55.5 ± 45.3 dd, 3 T, seq CUBE and T2	6, 12 and 24	Volume WORMS	33 LTP, 22 LFC 21 MTP, 6 MFC 4 PAT, 1 TRO	77,80%	20.7% 13.8% 87.2%	Osteochondral
Helito [42]	Retrospective	101, 79 M, 22 F, 33	≤ 3 weeks, 1.5 and 3.0 T, seq T1, T2 and PD	NR	NR	NR	NR	NR	NR
Berger [6]	Retrospective	220, 148 M, 72 F, 34 (16–71)	≤ 8 weeks, 1.5 T, seq FS and T2 FSE	NR	Volume WORMS	48 LTP, 44 LFC 43 MTP, 5 MFC 7 PAT	NR	NR	NR
Wang [93]	Cross sectional	130, 85 M, 45 F, (18–40)	NR, 1.5 and 3.0 T, seq T1 and PD FS SE	24 and 36	Volume	130 LTP, 130 LFC 130 MTP, 130 MFC	NR	NR	NR
Lattermann [57]	Prospective	81, NR, 35	NR, NS, seq T1, T2 and PD	24 and 72	Volume Costa-Paz	76 LTP, 66 LFC 46 MTP, 20 MFC	100%	NR	LM Osteochondral
Thomas [85]	Descriptive laboratory study	35, NR, 20	NR, 3 T, seq PD and FS	NR	Area	12 LTP, 12 LFC	NR	NR	NR
Driban [25]	Cross sectional	121, 89 M, 32 F, 26 (18–35)	≤ 4 weeks (19 ± 6.5), 1.5 T, seq DETSE	NR	Volume	116 LTP, 101 LFC 101 MTP, 64 MFC	96%	NR	Depression fracture
DePhillipo [20]	Case series	50, 33 M, 17 F, 30 (14–61)	NR, 1.5 and 3.0 T, seq PD, FS and T2	NR	NR	36 MTP	76%	NR	NR
Temponi [82]	Retrospective	162, NR, NR	≤ 1 week, 1.5 T, seq NS	NR	NR	24 LTP, 24 LFC 15 MTP, 9 MFC	75%	NR	Superior popliteomeniscal fascicle
Ali [2]	Retrospective	25, 15 M, 10 F, 30 (13.5–55)	≤ 24 dd (0–10 mm), 1.5 T, seq PD	NR	NR	4 LTP, 2 LFC 4 MTP, 2 MFC	60%	NR	LCL MCL

The studies are analysed for type of study, number of initial patients, mean (range) age, sex, follow-up time in months, edema distribution, prevalence, progression and correlation with other joint lesions. Some data discrepancies between overall population and subgroups are due to lack of patient details in some of the studies

*NR* not reported, *MFC* medial femoral condyle, *LFC* lateral femoral condyle, *MTP* medial tibial plateau, *LTP* lateral tibial plateau, *TRO* trochlea, *PAT* patella, *LCL* lateral collateral ligament, *MCL* medial collateral ligament, *LM* lateral meniscus, *MM* medial meniscus, *ALL* anterolateral ligament, *ICRS* International Cartilage Research Society, *MOAK* MRI Osteoarthritis Knee Score, *WORMS* Whole-Organ Magnetic Resonance Imaging Score

This systematic review revealed heterogeneous MRI sequences and assessment strategies. Bone bruise was quantified in 43/83 studies with the following approaches: scoring systems were used in 9/43 articles, including WORMS, Costa-Paz, ICRS, Lynch, Beattie and Colleagues score and MOAK, while different parameters such as area/volume of the region of interest (either with absolute or percentage values), depth, signal intensity, distribution and diameter were used as criteria in 42/43 cases to quantify bone bruise. These articles analysed heterogeneous populations, for a total of 10,047 patients, including 2,675 females and 4,665 males (in 11 studies sex was not specified) with different sport participation (four articles focusing on athletes, the others on patients with various activity levels). Age ranged from children (only one study), to young adults and to senior patients (5–81 years).

### Bone bruise prevalence and progression

**Fig. 3:**
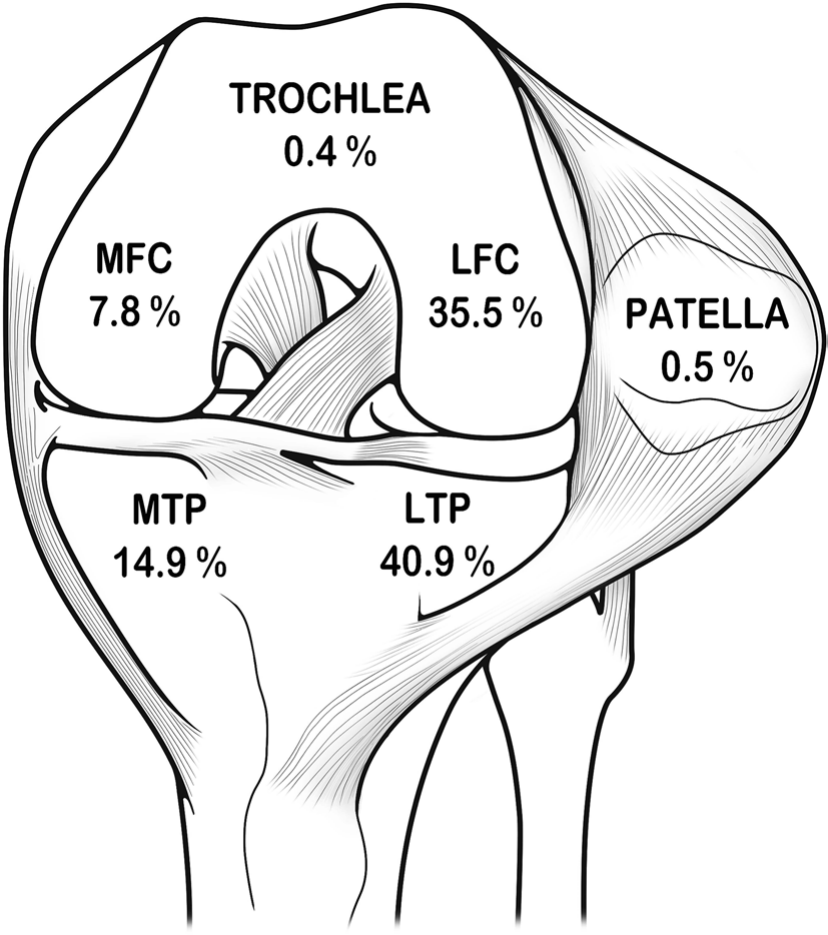
Percentage of bone bruise distribution in the affected anatomic bone locations. *LTP* lateral tibial plateau, *LFC* lateral femoral condyle, *MTP* medial tibial plateau, *MFC* medial femoral condyle

Prevalence of bone bruise ranged from 8 to 98% (reported in 40/83 papers), being higher in the most recent papers (78% in the last 10 years vs. 62% in previous papers). Most of the studies also investigated its distribution in the joint compartments, showing a higher prevalence in the lateral side of the knee (52/55), with lateral tibial plateau (31/43) being the most commonly affected site (Fig. 3). The evaluation of progression, investigated in 20 studies (seven retrospective, with heterogeneous follow-ups from 2 weeks to 13 years), documented a wide time range: from series showing complete resolution at 2 months, to others documenting persistence of subchondral marrow changes in 65% of the cases at 1 year, or even an increase of bone bruise in one-third of the patients over time.

Some factors were reported to influence frequency, distribution, and progression of bone bruise. Female sex, high BMI, complete vs. partial ACL tears and combined lesions were correlated to higher prevalence, while specific distribution patterns were influenced by injury mechanism, such as pivoting (more lateral), hyperextension (more anterior), motor vehicles accident and patellar dislocation (more anterior with patella involvement), as well as by gender and age (female and older patients presented more lateral lesions). Finally, progression was also influenced by some factors, with slower resolution in the presence of osteochondral lesions and after ACL reconstruction compared to more conservative treatments.

### Impact of bone bruise on joint lesion severity and progression

The severity of joint lesions, investigated in 30/83 studies, was correlated to the presence of bone bruise in 26/30 studies. The most affected tissue was cartilage: osteochondral lesions were reported to correlate significantly in 11/13 studies, ranging from 59% to more than 80% of patients (80% in the lateral tibial plateau or 94% in the lateral femoral condyle) affected by bone bruise; this was followed by meniscus lesions and, less frequently, by collateral ligaments and presence of fractures. Some reports also suggested a possible correlation with other lesions, such as those involving the anterolateral ligament, superior popliteomeniscal fascicle, as well as more abundant and slower resolving effusion. The rim sign, with anteromedial bone bruise distribution, was reported to be associated with greater joint derangement. Moreover, the presence of a > 1.5 mm notch sign, a bone depression due to more frequent impaction at the lateral femoral condyle after pivoting lesions, was reported to be associated with cartilage lesions and lateral meniscus tears. Finally, few studies evaluated articular samples showing softening, fissuring, with degeneration of chondrocytes and loss of proteoglycans, together with necrosis of osteocytes and empty lacunae in subchondral bone, as well as elevation of COMP degradative fragments, both at cartilage and synovial fluid level. Homeostatic alterations were also supported by changes of synovial fluid, which presented a higher presence of glycosaminoglycans. The impact of bone bruise on joint damage over time was explored, showing (4/8 papers) a correlation of bone bruise with persisting and progressive damage of the articular surface, suggesting early OA development [28].

Factors influencing lesion severity of joints presenting bone bruise were found in 20 studies, the most frequent being higher bone bruise size and severity, followed by taller patients and higher BMI. A larger bone bruise was also correlated with osteochondral lesion progression.

### Influence of bone bruise on the clinical outcome

Papers evaluating the influence of bone bruise on clinical outcome (19/83) studied 2822 patients, with a follow-up ranging from 1 week to 13 years. Several methods were used: subjective scoring systems, such as KOOS, Tegner, IKDC, SF-36; ADL, Lysholm, Noyes, and VAS, and other evaluation methods including ROM, clinical examination, and gait analysis.

Among these studies, five focused on baseline clinical findings, two of them showing a correlation of bone bruise with higher pain and laxity, especially in case of bone bruise with higher volume and at the medial side. Four studies focused on short-term recovery and documented a longer time to reach normal ROM and non-antalgic gait before ligament reconstruction, with a lower clinical outcome for up to 6 months, especially in case of larger size and medial side bone bruise distribution. Ten studies explored the mid-/long-term outcome: only one was able to document the influence of bone bruise on the mid-term clinical outcome, showing a lower return to sport after ACL reconstruction in joints presenting bone bruise at baseline MRI.

Finally, factors found to influence clinical findings were associated chondral lesions and osteochondral fractures, as well as bone bruise severity, location (lateral distribution with higher instability and ROM limitation, medial distribution with higher pain) and persistence over time; bone bruise detected at MRI performed more than 3 months after trauma was suggestive of a more difficult return to full activity recovery.

## Discussion

The most important finding of this systematic review of the literature is that bone bruise in ACL lesions is a frequently detected MRI finding that entails a more severe joint damage affecting joint degenerative progression.

Several articles have been published over the past 30 years and the interest on this topic is still growing, with an increasing number of studies in the recent past. Nonetheless, the contribution of the existing literature is limited, as most of the findings are accompanied by still open questions, which will be addressed in the following paragraphs. The first factor hindering the possibility to effectively summarize the study results is the lack of a common language in the literature. In fact, besides the overall accepted definition of bone bruise, when looking at lesion assessment and description, the literature showed no common strategy. The sequences used differed among studies, and half of the authors did not even describe the MRI findings observed. Moreover, those who aimed at further assessing the presence of bone bruise, applied heterogeneous methods relying on different grading systems or quantifying area or volume in the affected compartment in either absolute or relative values. The complexity of this scenario is further increased by the heterogeneity of the populations analysed, as well as outcomes and follow-up times investigated. In this light, the evidence on each specific aspect (prevalence, natural history and impact on joint and outcome) is often driven only by few low-quality studies, which explains the current persisting effort of physicians and researchers to further explore the role of bone bruise in ACL lesions.

Prevalence of bone bruise in the MRI of patients affected by ACL lesions has been investigated in most of the selected studies, showing a wide range from 12 to 96%. This heterogeneity can be explained by several factors, such as the differences of the analysed populations in terms of bone bruise joint distribution, mechanism of trauma, age, sex, activity and BMI. Among all, the main factor was the resolution time of the abnormal MRI signal, which makes the presence of bone bruise strongly related to the time passed from injury to MRI examination. To this regard, it is also interesting to observe how the reported prevalence increased in the past few years, which could be explained by different patients included but, possibly, also by the evolution of the MRI technology and sequences. While earlier reports tended to show a swift complete resolution, more recent findings show persistence or even increase of MRI abnormality over time [36]. However, if from one side, modern MRI can allow a more in-depth study of tissue alterations compared to earlier studies; on the other hand, the clinical significance of this more subtle, but now detectable changes, still remains controversial [62].

This systematic review evidenced several questions remaining still open, but at the same time it showed an increasing awareness on the importance of bone bruise. The attention on this matter can be better understood looking at the impact of ALC lesions on society: ACL reconstruction is one of the most common procedures in orthopaedics [61]. Associated injuries and earlier onset of degenerative changes influence the affected knee: high rate (from 10 to 90%) of osteoarthritis development after ACL injury has been reported despite continuous efforts to optimize ACL treatment [69]. This has prompted researchers to look at possible factors affecting the evolution of joint degeneration.

A correlation between bone bruise and cartilage lesions has been demonstrated, and it is well acknowledged that the presence of cartilage lesions increases with the time elapsed between ACL rupture and reconstruction: chondral lesions may increase the chances of osteoarthritis development, even after ACL surgical repair [30]. Even though in most cases normal cartilage is initially found during arthroscopy, the osteochondral unit absorbs compression forces during impaction, and this could cause a double long-term mechanism of damage of the articular surface. Cartilage metabolism may be significantly affected, with long-term consequences [24]. Moreover, abnormality [86] of subchondral bone may precede and favour cartilage destruction, since the rigid callus resulting from bone fracture may cause cartilage to absorb more of the load force, with abnormal stresses leading to a progressive degeneration of the articular surface [62]. In ACL reconstructed knees, the cartilage overlying the area of bone bruise presents signs of damage with altered extracellular matrix: cartilage evaluated at 12 months’ follow-up with recent MRI sequences showed elevated T1ρ values compared to the surrounding tissue, thus suggesting that despite the resolution of abnormal bone signal, cartilage lesions persist [84]. These imaging data have been confirmed by histological data, and the analysis of joint samples documented an alteration of the entire joint homeostasis [87].

Bone bruise in ACL injury is correlated with osteochondral lesions that can act as a catalyst for osteoarthritis even after a successful reconstruction. In this light, it appears logical to suppose that such an important trauma causing these deleterious consequences on joint tissues might also affect clinical prognosis. However, results on this matter are controversial [32, 67]. The lack of evidence on the correlation between the presence of bone bruise at MRI after trauma and the long-term effect on the joint with the reconstructed ACL may be explained by several factors, starting from the lack of long-term studies, which could better detect the effect on the joint of the cascade started with the initial trauma [32]. Moreover, current classification systems contribute little towards the understanding of the underlying pathology defined as bone bruise. Relatively less severe trauma causes marrow edema without injury to cells and subchondral bone architecture. However, when the extent of trauma is bigger, trabecular fractures and haemorrhage are seen together with edema, but current MRI sequences and bone bruise definition do not help in distinguishing between these two patterns [62]. Similarly, it is not always easy to distinguish between bone bruise involving only the marrow with occult fractures not breaching the adjacent cortex, and those involving the osteochondral surface. Moreover, factors predictive of subsequent trabecular fracture development have not been identified yet. In fact, even bone bruise without cortical disruption may represent a region of bone at increased risk for the subsequent development of insufficiency fractures, if bone is not adequately protected during trabecular healing.

Some efforts have been made to identify predicting factors, such as the importance of the localization of the imaging finding and its evolution pattern, with resolution proceeding from periphery to joint margin (opposite to lesions proceeding toward the centre of bone bruise lesion) being suggested to be associated with osteochondral injuries. However, these correlations are mainly related to sporadic evidence [19, 34, 55, 90]. In fact, most of the studies do not address the different evolution patterns according to possible influencing variables, but rather report overall outcomes on heterogeneous populations. The lack of focus on specific patient populations is another aspect that may hinder a better understanding of the long-term clinical impact, since joint tissue damage can variably affect patients with a different activity level [32].

Prospective studies are needed to look at the natural history of bone bruise and at identifying factors affecting its radiological and clinical course. At the moment, clinical management remains complicated, both because it is very difficult to identify specific clinical signs and symptoms due to concomitant knee damages (soft tissue lesions and effusion), and because of the lack of correlation between symptoms and imaging findings. Imaging resolution is largely delayed compared to clinical symptomatology [4], which currently guides clinical management. The understanding of whether and how to protect cartilage (by rest from weight bearing) during initial treatment, when cartilage lacks support from bruised bone [21, 54, 55], or the development of treatments to address both stiffer long-term tissue repair and altered homeostasis [3, 22, 31], would likely contribute to overall better results.

During ACL injury, the entire joint undergoes a high-energy trauma, which may alter joint homeostasis and long-term prognosis. In this light, even if no statistical analysis was feasible due to the heterogeneity of the included studies, this systematic review provides evidence-based insights to understand the significance of this articular derangement, which can be of clinical relevance for the orthopaedic surgeon when dealing with patients affected by bone bruise following ACL injury, by underlining that this MRI finding may play an important role in the joint derangement affecting the outcome ACL reconstruction surgery. Future research should aim at better understanding clinical significance, factors predicting resolution or long-term consequences to the affected joint and patient prognosis and, finally, at identifying strategies to restore the overall joint homeostasis rather than just the ligament lesion. This would optimize the management of ACL-injured patients with better long-term results.

## Conclusion

Bone bruise has a high prevalence, with distinct patterns related to the mechanism of injury, and its presence and persistence have been correlated to a more severe joint damage, which may affect the degenerative progression of the entire joint, with recent evidence suggesting possible effects on the long-term clinical outcome. However, prospective long-term studies are needed to better understand the natural history of bone bruise, identifying prognostic factors and targets of specific treatments that could be developed in light of the overall joint derangements accompanying ACL lesions.
